# Prognostic significance of miR-1268a expression and its beneficial effects for post-operative adjuvant transarterial chemoembolization in hepatocellular carcinoma

**DOI:** 10.1038/srep36104

**Published:** 2016-10-31

**Authors:** Yun-Long Lu, Jin-Guang Yao, Xiao-Ying Huang, Chao Wang, Xue-Min Wu, Qiang Xia, Xi-Dai Long

**Affiliations:** 1 Department of Pathology, the Affiliated Hospital of Youjiang Medical University for Nationalities, Baise 533000, P.R.China; 2 Department of Medicine, the Affiliated Hospital of Youjiang Medical University for Nationalities, Baise 533000, P.R.China; 3 Department of Liver Surgery, Ren Ji Hospital, School of Medicine, Shanghai Jiao Tong University, Shanghai 200127, P.R.China

## Abstract

Our recent investigation has shown that the variables of microRNA-1268a may involve in hepatocellular carcinoma (HCC) tumorigenesis. Here, we attempted to identify the prognostic significance of microRNA-1268a expression in tumor tissues by a retrospective analysis in 411 patients with HCC, and analyze its effects on post-operative adjuvant transarterial chemoembolization (TACE) improving HCC prognosis. All cases received tumor resection or tumor resection plus post-operative adjuvant TACE as an initial treatment. Logistical regression analysis exhibited that microRNA-1268a expression was significantly correlated with tumor stage, tumor grade, tumor size, and microvessel density. Cox regression analysis showed that microRNA-1268a expression was an independent prognostic factor for HCC, and TACE treatment had no effects on prognosis of HCC patients with high microRNA-1268a expression. More intriguingly, TACE improved the prognosis of HCC patients with low microRNA-1268a expression. Functionally, overexpression of microRNA-1268a inhibited while its inhibitor enhanced doxorubicin-induced the death of cancer cells. These results suggest that microRNA-1268a may be an independent prognostic factor for HCC patients, and that decreasing microRNA-1268a expression may be beneficial for post-operative adjuvant TACE treatment in HCC.

Hepatocellular carcinoma (HCC) is a life-threating malignancy, accounting for more than 90% of primary liver cancer. This malignant tumor is the fifth most common cancer in men and the seventh in women. Because of its very poor prognosis resulting from metastasis and recurrence, it has been regarded as the third most common cause of death from cancers worldwide^1^. In the past decades, several new treatment options have been developed and recommended for patients with HCC^2^,^3^,^4^,^5^. Among these treatments, the transarterial chemoembolization (TACE) is reported to be an effective treatment for advanced-stage HCC patients, especially for those cases with 3- to 5-cm tumors^5^,^6^. However, increasing evidence has shown that TACE treatment may display different therapeutic effects on HCC patients with different genetic profiles^7^,^8^,^9^,^10^,^11^,^12^. Furthermore, the long-term survival outcomes of patients managed with TACE do not appear fully satisfactory^13^,^14^,^15^. Therefore, it is important to discern what kinds of HCC and genetic profiles can benefit from post-operative TACE treatment.

MicroRNAs are a class of small non-coding single-stranded RNAs with about 20 nucleotide sequences, and are formed from the sequential processing of primary transcripts by Drosha and Dicer RNase enzymes^16^,^17^. Through regulating gene expression, they functionally involve in not only cell proliferation, differentiation, and apoptosis, but also metabolism, physiological timing, and hormone secretion^16^,^18^,^19^,^20^. Until now, more than 2000 microRNAs have been identified; parts of them (such as microRNA −624, microRNA −24, microRNA-101, and so on) have shown to be able to act as valuable prognostic factors and potential therapeutic targets for HCC^11^,^18^,^19^,^20^,^21^,^22^. Among these microRNAs, microRNA-1268a (miR-1268a), which is an important abundant microRNA encoded by MIR1268A gene and functionally involves in embryogenesis and cell differentiation^23^, is particular concern in our research projects. Our previous data from a large molecular epidemiological investigation have exhibited that the genetic variables in the seeding region of miR-1268a were correlated with tumor angiogenesis and may involve in the carcinogenesis of HCC^24^. This suggests a possible relationship between miR-1268a and the prognosis of HCC characterized by rich blood vessels. Therefore, we continued to investigate the possible prognostic significance of miR-1268a expression for HCC patients and possible value for the selection of post-operative adjuvant TACE treatment in this study.

## Materials and Methods

### HCC patients

This study was a hospital-based retrospective study, and the study protocol was approved by the Institutional Ethics Committee of Youjiang Medical University for Nationalities, and was carried out in accordance with the approved guidelines (No. 20041225). The patients with HCC were recruited in the affiliated hospitals of Youjiang Medical University for Nationalities and Guangxi Medical University. The inclusion criteria on cases are as follows: *a.* HCC confirmed by histopathological examination; *b.* the objective of the study was understood and informed consent was provided; *c.* the ability to complete the necessary investigations and questionnaires; *d.* cases underwent tumor resection or tumor resection plus post-operative TACE as an initial therapy according to Chinese Manage Criteria of HCC (solitary or multiple tumors mainly located in one lobe of the liver; no extrahepatic metastases; Child-Pugh A-stage liver function; and no contraindication for laparotomy)^25^, but not treatment with chemotherapy or radiotherapy before surgical operative treatment; and *e.* 5-year follow-up completed and with available fresh cancerous tissue specimens and clinical data. The exclusion criteria included: *a.* cases with HCC but not confirmed by histopathological examination; *b.* cases with history of chemotherapy or radiotherapy treatment before surgical operative treatment; and *c.* cases rejected, dropped out, or lost information. According to aforementioned inclusion and exclusion criteria, a total of 411 HCC cases (representing 98.5% of eligible cases), including 157 patients previously studied^11^,^12^,^21^, were included for the present study from January 2006 to December 2009.

### Samples and data collection

After written consent was obtained, surgically removed tumor samples of all patients with HCC at the starting point of the initial treatment were collected for analyzing miR-1268a expression levels. Demographic information (including gender, age, ethnicity, hepatitis B virus [HBV] and hepatitis C virus [HCV] infection) and clinical pathological data (including cirrhosis, tumor size, tumor grade and stage, and treatment information) were collected in the hospitals using a standard interviewer-administered questionnaire and/or medical records by a Youjiang Cancer Institution staff member. In this study, those anti-HCV positive and hepatitis B surface antigen (HBsAg) positive in their peripheral serum were defined as groups infected with HCV and HBV. Tumor grade and stage were defined according to Edmondson and Steiner (ES) grading system^26^ and the Barcelona Clinic Liver Cancer (BCLC) staging system^6^, respectively. To analyze, tumor grade was divided into two subgroups: low grade (ES-I and -II) and high grade (ES-III and -IV), in this study. Liver cirrhosis was evaluated by pathological examination.

To elucidate the role of post-operative adjuvant TACE in patients with different levels of miR-1268a expression, all corresponding TACE treatment information was also collected. TACE was performed as a part of the initial treatment procedure (starting 4 weeks after tumor resection). The inclusion criteria for TACE are as follows: *a.* a pathologically diagnosed HCC with BCLC stage B or C; *b.* compensated cirrhosis with Child-Pugh stage A or no cirrhosis; *c.* multiple tumors more than 5 cm or tumor involving a first or second branch of the portal or hepatic veins; *d.* the tumor with multiple lesions localized in one lobe of liver, or the main tumor localized in one lobe only with a small solitary lesion in contralateral lobe, or tumor involving a first or second branch of the portal or hepatic vein, which could be safely resected without grossly remaining tumors, and the patient was judged to have well preserved liver function to survive the operation; *e.* cases underwent partial hepatectomy, and agreed to post-operative adjuvant TACE treatment; and *f.* no contraindication for TACE. The exclusion criteria included: *a.* patients with non-HCC on postoperative histopathological examination, serious concurrent medical illness, intractable ascites, tumor recurrence within 4 works after the operation, and women who were pregnant or breastfeeding; *b.* cases rejected, dropped out, or lost information; *c.* cases with contraindication for TACE; and *d.* cases with history of chemotherapy or radiotherapy treatment before surgical operative treatment. In the present study, TACE consisted of an injection containing a mixture of chemotherapeutic agents and lipiodol followed by embolization with gelatin foam or polyvinyl alcohol until complete stasis was achieved in the tumor-feeding vessels. The chemotherapeutic agents used concurrently included Doxorubicin and Cisplatin.

For survival analysis, all HCC cases were followed up as described in our previous studies^12^,^21^,^27^,^28^. The last follow-up day was set on December 31, 2015, and survival status was confirmed by means of patient or family contact and clinic records. In this study, the duration of the duration of overall survival (OS) was defined as from the date of the initial treatment completion to the date of death or last known date alive; whereas tumor recurrence-free survival (RFS) was defined as from the date of the initial treatment completion to the date of tumor recurrence or last known date alive^12^,^21^,^27^,^28^.

### MiR-1268a expression assay

The level of miR-1268a expression in cancerous tissues was analyzed using our previously published TaqMan quantitative reverse transcription-PCR technique^24^. In this study, the relative amount of miR-1268 to internal control U6 was calculated as 2^−ΔCt^ method, where ΔCt = (Ct_miR-1268a_ − Ct_U6_). For analysis, miR-1268a expression levels were divided into two groups: low expression group (2^−ΔCt^ ≤ 2.00) and high expression group (2^−ΔCt^ > 2.00) according to the average value among HCC cases.

### The micro-vessel density (MVD) evaluation

In the present study, the angiogenesis of cancerous tissues was assessed by the micro-vessel density (MVD) as previously ascribed^21^. Briefly, vessels were stained by CD31 (cat#2011101101, Gene Tech (Shanghai) Company Limited, Shanghai, China) and counted in the cancerous regions over five fields (at X200 magnification) in each slide. The MVD was defined as positive when the average value of the three readings >50^21^.

### Cell culture and transfection

The SMMC-7721 cells (a kind of HCC cell line) were purchased from Cell Resource Center of Shanghai Institute for Biological Sciences, Shanghai, China. Cells were cultured in Dulbecco’s Modified Eagles Medium (HyClone, Thermo Fisher Scientific (China) CO., Ltd, Shanghai) containing 10% fetal bovine serum (Gibco-Invitrogen Corp., Carlsbad, CA) in atmosphere of 5% CO_2_ at 37 °C using standard techniques. Cells were transfected with miR-1268a mimics (GenePharma, China), its inhibitor (GenePharma), or normal saline using Invivofectamine^®^ 12.0 Reagent (cat# 1377501, Life) according to the manufacturer’s instructions. In this study, transfection efficacy was elucidated as the ratio of transfected cells detected by the LV200 system to total cells obtained from three different regions at random, and was about 80%.

### Cell sensitivity assay

The sensitivity of SMMC-7721 cells to doxorubicin was elucidated by the half-maximal inhibitory concentration (IC50) using a cell counting kit (CCK-8) assay (cat# CK04, DojindoCorp., Japan) according to the manufacturer’s instructions. Briefly, a total of 5000 cells were seeded each well in a 96-well plate and transfected, followed by treatment with doxorubicin at 15 different concentrations (0.01–40 μM) (48 hours after transfection). After 36 hours of treatment, the CCK-8 solution was added to the well and incubated for 2 hours at 37 °C. Then, the absorbance of optical density (at 450 nm) was recorded, and IC50 values were calculated by nonlinear regression analysis using the GraphPad Prism software with Version 6.0 (GraphPad Software, Inc., San Diego, CA, USA).

### TUNEL assay

Cells were seeded in six-well plates for 24 hours, and then transfected with miR-1268a mimics or miR-1268a inhibitor. Forty-eight hours after transfection, cells were treated with doxorubicin (1.25 μM). After treatment for 36 and 48 hours, the cells were all harvested and analyzed by TUNEL staining using an *in situ* cell death detection kit (Roche, Mannheim, Germany) in combination with 4,6-diamino-2-phenyl indole staining. TUNEL-positive cells were counted in at least 300 cells in randomly chosen fields. The data were expressed as a percentage of TUNEL positive cells to total cells.

### Statistical analysis

All analyses were performed with the statistical package for social science (SPSS) version 18 (SPSS Institute, Chicago, IL, USA). The differences of age, race, gender, and liver function between groups were compared using the χ2 test or one-way ANOVA with Bonferroni corrections. Non-conditional logistic regression was used to evaluate odds ratios (ORs) and 95% confidence intervals (CIs) for the effects of miR-1268a expression on the pathological features of HCC. Kaplan–Meier survival analysis (with the log-rank test) was used to evaluate the association between miR-1268a expression and HCC prognosis. Hazard ratios (HRs) and 95% CIs for miR-1268a expression were calculated from univariate and multivariate Cox regression model. In this study, a *P*-value of less than 0.05 was considered statistically significant.

## Results

### MiR-1268a expression correlated with clinic-pathological features of HCC

Table 1 showed the clinic-pathological data of the cases and their association with miR-1268a expression. The present study comprised of 411 HCC patients with 278 (67.6%) males and 133 (32.4%) females. The mean age was 47.8 ± 10.3 years. Among these patients, more than 70% of cases were infected by HBV, and most of them had liver cirrhosis. All of these cases underwent either tumor resection or tumor resection plus post-operative adjuvant TACE as an initial treatment according to Chinese Manage Criteria of HCC^25^. During the follow-up period of these patients, 244 faced tumor recurrence with 40.9% of the 5-year RFS rate, and 256 died with 43.5% of the five-year OS rate.

To explore the association between miR-1268a expression and clinic-pathological features of patients with HCC, we tested the levels of miR-1268a expression in cancerous tissues using TaqMan-PCR method. Among these patients, 230 (56.0%) featured low miR-1268a expression. Results from non-conditional logistic regression analysis showed that decreasing miR-1268a expression was significantly related to larger tumor size (OR = 1.96), tumor dedifferentiation (OR = 2.78), higher tumor stage (OR = 4.17), and higher MVD (OR = 2.56), but not to other features.

### Univariate analyses identified miR-1268a expression as a significant prognostic predictor for survival of patients with HCC

Although we previously showed that the mutation at seeding region of miR-1268a was significantly correlated with poor OS and RFS of patients with HCC patients^24^, it remains to be addressed whether miR-1268a expression is a prognostic marker for HCC patients. For this purpose, we performed the univariate analyses to test the associations of relatively expressed miR-1268a levels and standard variables with OS and RFS of HCC cases. As shown in Table 2, genders, age, ethnicity, smoking and drinking status, HBsAg, anti-HCV, AFP levels, and tumor grade were not associated with survival; while miR-1268a expression was a significant prognostic marker [HR (95% CI) = 3.23 (2.44–4.17) for OS and 3.70 (2.78–4.76) for RFS, respectively], as well as MVD, BCLC stage, and tumor size. This suggests that increasing levels of miR-1268a might improve survival of cases with HCC.

### MiR-1268a expression was an independent factor for HCC prognosis

**Figure 1:**
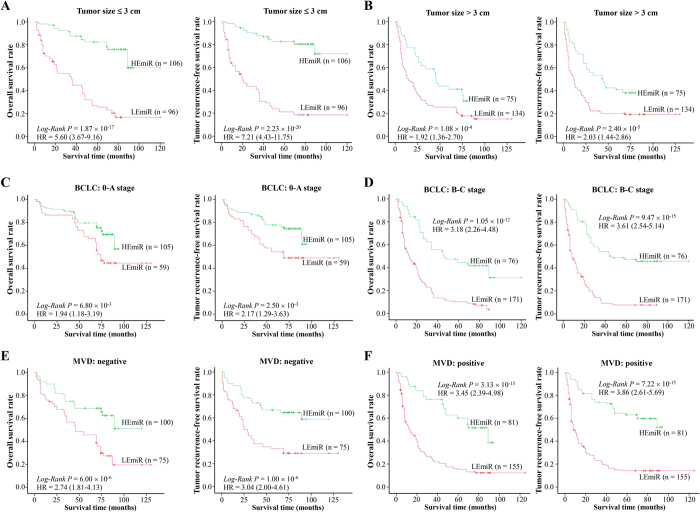
The miR-1268a expression significantly affected hepatocellular carcinoma (HCC) prognosis in the strata analysis of tumor size, stage, and micro-vessel density (MVD). (**A,B**) The effects of miR-1268a expression on overall survival (OS, left) and tumor recurrence-free survival (RFS, right) in strata of tumor size. (**C,D**) The effects of miR-1268a expression on OS (left) and RFS (right) in strata of tumor stage. (**E,F**) The effects of miR-1268a expression on OS (left) and RFS (right) in strata of MVD. Cumulative hazard function was plotted by Kaplan-Meier’s methodology, and *P* value was calculated with two-sided log-rank tests. Relative hazard ratios (HRs) and corresponding 95% CIs of low miR-1268a expression (compared with high expression) were calculated using multivariable cox regression model (including all significant variables). *Abbreviation.* BCLC, the Barcelona Clinic Liver Cancer staging system of HCC; HEmiR, high expression of miR-1268a; LEmiR, low expression of miR-1268a.

Because some clinic-pathological features such as tumor stage, tumor size, and MVD were also associated with poor clinical outcome, we aimed to determine whether reduced OS and RFS observed in patients with low expression of miR-1268a was an indirect reflection of association between decreasing expression of miR-1268a and these clinicopathological markers or, alternately, whether decreasing miR-1268a expression might be an independent prognostic factor. To answer this, we first accomplished series stratified analyses based on above-mentioned features (Fig. 1). Results exhibited miR-1268a expression significantly modified the prognosis of cases with HCC among different strata. Multivariate cox regression analysis (including all known clinic-pathological variables) was next performed to determine whether miR-1268a expression was an independent predictor of patients with HCC (Table 3). The results showed that tumor size (HR 1.90, 95% CI 1.44–2.49), MVD (HR 1.80, 95% CI 1.37–2.38), and tumor stage (HR 3.70, 95% CI 2.70–5.06) as well as miR-1268a expression (HR 2.44, 95% CI 1.82–3.23) were determined as independent prognostic factors for OS. Risk role was also found in the RFS analysis. Taken together, these results suggested that miR-1268a could be used as an independent prognostic marker for HCC.

### MiR-1268a expression differentially affects therapeutic effects of TACE intervention in HCC patients

**Figure 2:**
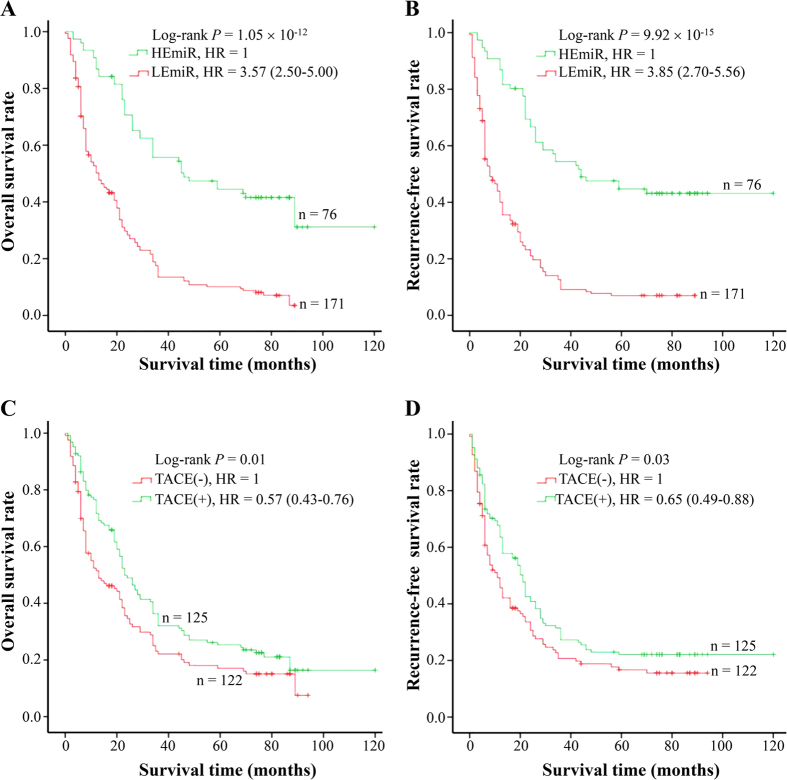
The effects of miR-1268a expression and TACE on HCC prognosis in 247 cases with BCLC B- and C-stage HCC. According to the average expression in cancerous tissues, the levels of miR-1268a expression were divided into two groups: low expression group (relative level ≤2) and high expression group (relative level >2). MiR-1268a expression was associated with overall survival (**A**) and tumor reoccurrence-free survival (**B**) of HCC. TACE treatment was also related to HCC prognosis (**C**,**D**). Cumulative hazard function was plotted by Kaplan-Meier’s methodology, and *P* value was calculated with two-sided log-rank tests. Relative hazard ratio (HR) and corresponding 95% CI of high miR-1268a expression (compared with low expression) and TACE treatment (compared with negative TACE) was calculated using multivariable cox regression model (including all significant variables). *Abbreviation.* HEmiR, high expression of miR-1268a; LEmiR, low expression of miR-1268a.

**Figure 3:**
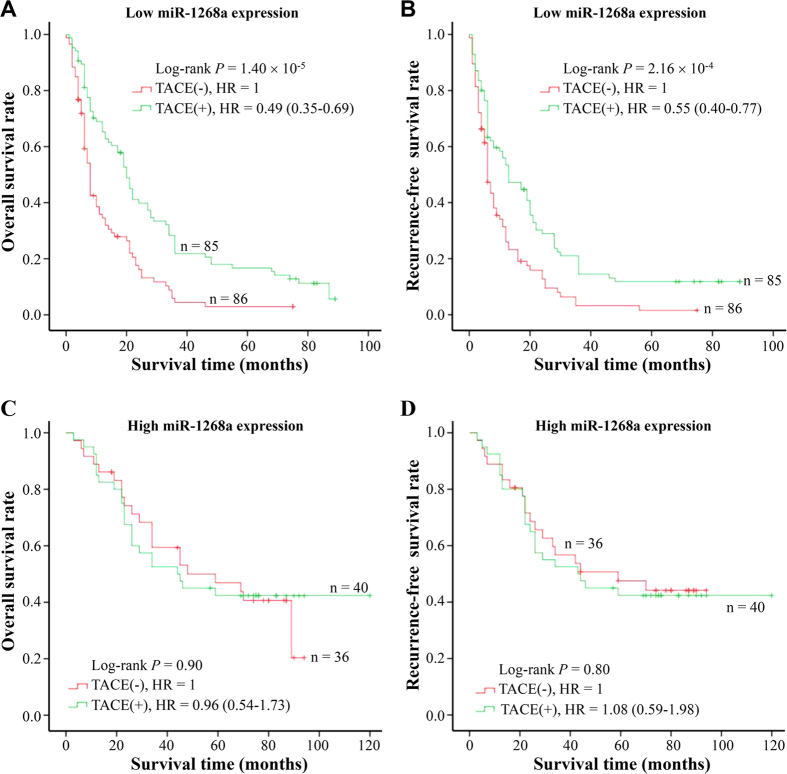
Survival analysis of TACE treatment in strata of miR-1268a expression. Among HCC cases with low miR-1268a expression, TACE treatment affected HCC overall survival (**A**) and tumor reoccurrence-free survival (**B**); but not among patients with high miR-1268a expression (**C**,**D**). Cumulative hazard function was plotted by Kaplan-Meier’s methodology, and *P* value was calculated with two-sided log-rank tests. Relative hazard ratio (HR) and corresponding 95% CI of TACE treatment (compared with negative TACE) was calculated using multivariable cox regression model (including all significant variables).

Given that miR-1268a expression is reversely associated with MVD of HCC, we questioned whether miR-1268a expression modified the therapeutic effects of TACE. To answer it, we investigated the correlation between miR-1268a expression and the therapeutic effects of TACE on cases with BCLC B-C stage HCC (n = 247) using a retrospective analysis (Table 4, Figs 2 and 3). Among these subjects, 125 received tumor resection plus post-operative adjuvant TACE as an initial treatment and were defined as TACE group. Others who only underwent tumor resection as an initial treatment were defined as non-TACE control group (n = 122). We did not observe substantial differences between TACE and control group in terms of the distribution of clinic-pathological features (Table 4). However, both miR-1268a expression (Fig. 2A,B) and TACE treatment (Fig. 2C,D) modified the survival of HCC patients. More intriguingly, the stratified analysis based on the levels of miR-1268a expression showed that TACE treatment significantly improved the OS and RFS among patients with low miR-1268a expression in cancerous tissues (Fig. 3A,B), but not among those with high miR-1268a expression (Fig. 3C,D). Taken together, these results suggest that miR-1268a expressions might be able to modify the effects of TACE treatment to predict the survival of HCC patients.

### MiR-1268a expression affected the effects of doxorubicin on HCC

**Figure 4:**
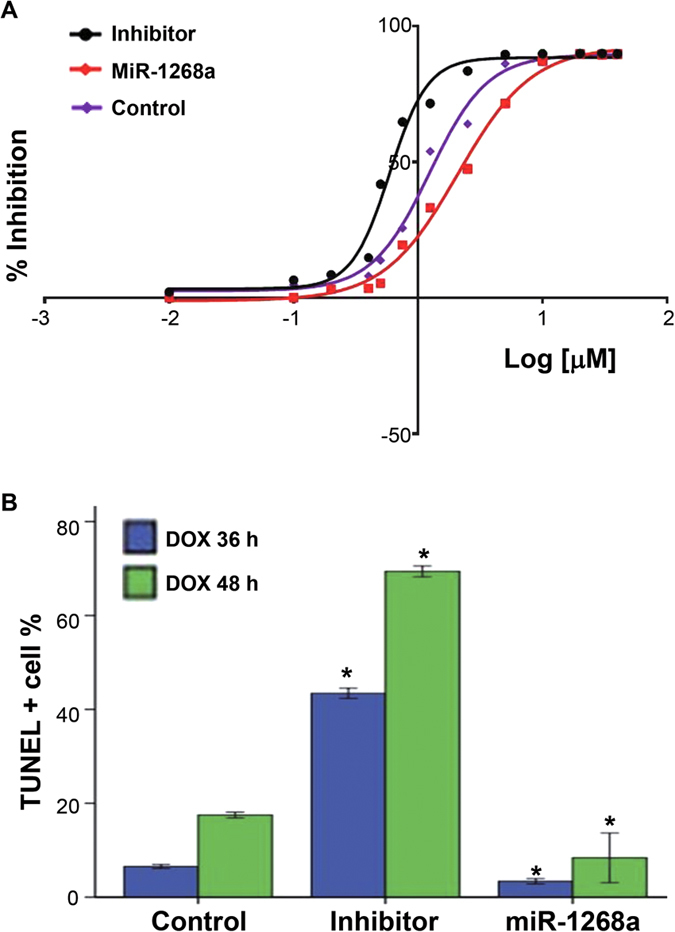
The miR-1268a expression modified effects of doxorubicin treatment on HCC cells SMMC-7721 *in vitro*. SMMC-7721 cells were transfected with normal saline (Control) or miR-1268a mimics (miR-1268a). (**A**) The sensitivity of cells to doxorubicin was evaluated by the half-maximal inhibitory concentration. (**B**) TUNEL staining was used to analyze the doxorubicin-induced cell deaths. Data were analyzed using *t* test.

Based on the above-mentioned findings that TACE treatment was beneficial for these cases with low miR-1268a expression but not those with high expression, we hypothesize that miR-1268a expression can affect the sensitivity of HCC cells to doxorubicin, an important chemotherapeutic drug used in TACE treatment^29^. To address this, SMMC-7721 cells were transfected with miR-1268a mimics, its inhibitor, or normal saline, followed by the treatment with increasing concentrations (from 0.01 to 40 μM) of doxorubicin. Thirty-six hours later, CCK-8 assay showed that miR-1268a mimics significantly decreased sensitivity of SMMC-7721 cells to doxorubicin compared to normal saline control. The IC50 (95% CI) values of doxorubicin were 2.09 (1.92–2.27) vs. 1.24 (1.13–1.35) μM for miR-1268a mimics vs. normal saline (Fig. 4A). Reversely, the inhibition of miR-1268a increased sensitivity of cancer cells to doxorubicin, with an IC50 value 0.59 (0.55–0.64) μM. Furthermore, down-regulation of miR-1268a expression significantly increased doxorubicin -induced cell death in the SMMC-7721 cells; whereas overexpression of miR-1268a decreased this kind of cell death, as assessed by TUNEL assay (Fig. 4B).

## Discussion

On the basis of a relative large-scale retrospective study, we investigated the association between miR-1268a expression and the prognosis of patients with HCC. It was found that the decreasing levels of miR-1268a expression had a substantial association with the poor outcome of HCC (HR, 2.44 and 95% CI, 1.82–3.23 for OS, 2.86 and 2.08–3.85 for RFS, respectively). Interestingly, TACE treatment could significantly improve the survival for HCC patients with low miR-1268a expression, but not for those with high miR-1268a expression. This study represents the first report indicating the potential of miR-1268a expression as a prognostic indicator of HCC.

The miR-1268a is an important abundant microRNA encoded by the corresponding gene MIR1268A that maps to human chromosome 15q11.2 regions. This microRNA was primarily identified in human embryonic stem cells using the Illumina sequencing technology in 2008 by Morin, *et al*.^23^. Higher miR-1268a expression was observed in the human embryonic stem cells than differentiated cells from embryoid bodies, implying miR-1268a might play an important role in embryogenesis and cell differentiation^23^. In our previous report, we showed that the structural variable in the seeding region of miR-1268a was positively associated with clinic-pathological features of HCC (including tumor differentiation and tumor grade and stage), and might involve in the process of HCC tumorigenesis^24^. These early findings prompted us to investigate whether the dysregulation of miR-1268a expression correlated with these features. In the present study, we collected 411 HCC tissue samples from Guangxi Zhuang Autonomous Region, a high epidemic area of HCC in China^30^, and explore the possible effects of miR-1268a expression on HCC prognosis. We found that HCC patients with low miR-1268a expression in the cancerous tissues faced a significant poor OS and RFS compared with those with high expression of miR-1268a. Multivariate cox regression analysis showed this down-regulating miR-1268a expression increased 2.44-times death risk and 2.86-times tumor recurrence risk; moreover, this risk did not depend on the clinic-pathological change. These data suggested that miR-1268a expression might be an independent prognostic factor for HCC and that its abnormal expression could be used as a prognostic marker for HCC.

In this study, we stratified HCC patients with respect to different levels of miR-1268a expression and investigated the effects of TACE on HCC prognosis in different expression status. This was done primarily because our present results exhibited miR-1268a expression was reversely associated with MVD in the cancerous tissues of HCC, and might involve in tumor angiogenesis. TACE can improve the survival of HCC among these cases with low miR-1268a expression but not among those with high miR-1268a expression, suggesting miR-1268a expression could modify the sensitivity of tumor cells on TACE treatment. The following *in vitro* analysis proved the aforementioned hypothesis. Therefore, it is well known that miR-1268a might be a useful marker for the selection of TACE treatment.

However, the present study had several limitations. Only 411 HCC patients were enrolled for prognosis analysis of HCC. We would like to confirm the findings in a larger HCC patient population. Another important limitation was that we did not do functional assays to validate the involvement of miR-1268a in tumor tumorigenesis and angiogenesis. Although these cases with BCLC B- or C- stage HCC in this retrospective study underwent partial liver resection according to Chinese Manage Criteria of HCC^25^, new management strategies for HCC showed BCLC B-C stage patients were not suitable for operative resection^6^. Additionally, the study design was retrospective which was easy to be affected by information bias. Therefore, more detailed molecular pathogenesis analyses and more studies (including prospective studies) deserve elucidation based on the results from large samples.

In conclusion, this study is, to the best of our knowledge, the first report that describes miR-1268a expression in HCC cancerous tissues and its associations with HCC prognosis. Our results showed that miR-1268a can act as a biomarker for the prognosis of HCC patients and for predicting the therapeutic effectiveness of TACE on these patients. Furthermore, miR-1268a can modify sensitivity of HCC cells to chemotherapeutic drugs. Given that HCC is a highly fatal tumor^1^, these findings may have implications for screening and prevention.

## Additional Information

**How to cite this article**: Lu, Y.-L. *et al*. Prognostic significance of miR-1268a expression and its beneficial effects for post-operative adjuvant transarterial chemoembolization in hepatocellular carcinoma. *Sci. Rep.* **6**, 36104; doi: 10.1038/srep36104 (2016).

**Publisher’s note:** Springer Nature remains neutral with regard to jurisdictional claims in published maps and institutional affiliations.

## Figures and Tables

**Table 1:** The association between miR-1268a expression and clinic-pathological features of HCC.

Variables	Cases, n (%)	MiR-1268a expression, n (%)		OR (95% CI)	*P*_trend_
		High	Low		
Total	411 (100.0)	181 (100.0)	230 (100.0)		
Age (yrs)					
≤48	228 (55.5)	94 (51.9)	134 (58.3)	Reference	
>48	183 (44.6)	87 (48.1)	96 (41.7)	1.28 (0.87–1.89)	0.21
Sex					
Man	278 (67.6)	120 (66.3)	158 (68.7)	Reference	
Female	133 (32.4)	61 (33.7)	72 (31.3)	1.10 (0.72–1.67)	0.67
Ethnicity					
Han	219 (53.3)	98 (54.1)	121 (52.6)	Reference	
Zhuang	192 (46.7)	83 (45.9)	109 (47.4)	0.94 (0.64–1.39)	0.76
HBsAg					
Negative	103 (25.1)	52 (28.7)	51 (22.6)	Reference	
Positive	308 (74.9)	129 (71.3)	179 (77.4)	0.71 (0.45–1.11)	0.13
anti-HCV					
Negative	363 (88.3)	157 (86.7)	206 (89.6)	Reference	
Positive	48 (11.7)	24 (13.3)	24 (10.4)	1.32 (0.72–2.44)	0.37
Smoking status					
No	299 (72.7)	128 (70.7)	171 (74.3)	Reference	
Yes	112 (27.3)	53 (29.3)	59 (25.7)	1.20 (0.78-1.85)	0.40
Drinking status					
No	288 (70.1)	126 (69.6)	162 (70.4)	Reference	
Yes	123 (29.9)	55 (30.4)	68 (29.6)	1.05 (0.68–1.61)	0.83
AFP (ng/mL)					
≤20	149 (36.3)	68 (37.6)	81 (35.2)	Reference	
>20	262 (63.7)	113 (62.4)	149 (64.8)	0.90 (0.60–1.35)	0.63
Liver cirrhosis					
No	103 (25.1)	53 (29.3)	50 (21.7)	Reference	
Yes	308 (74.9)	128 (70.7)	180 (78.3)	0.66 (0.42–1.04)	0.07
Tumor size					
≤3 cm	202 (49.1)	106 (58.6)	96 (41.7)	Reference	
>3 cm	209 (50.9)	75 (41.4)	134 (58.3)	1.96 (1.32–2.94)	8.35 × 10^−4^
Tumor grade					
Low grade	230 (56.0)	121 (66.9)	97 (42.2)	Reference	
High grade	181 (44.0)	60 (33.1)	133 (57.8)	2.78 (1.85–4.17)	8.27 × 10^−7^
BCLC stage					
0-A	164 (39.9)	105 (58.0)	59 (25.7)	Reference	
B-C	247 (60.1)	76 (42.0)	171 (74.3)	4.17 (2.72–6.40)	5.44 × 10^−11^
MVD					
Negative	175 (42.6)	100 (55.2)	75 (32.6)	Reference	
Positive	236 (57.4)	81 (45.8)	155 (67.4)	2.56 (1.69–3.85)	6.00 × 10^−6^

**Table 2:** Prognostic factors of OS and RFS for 411 patients with HCC by univariate analyses.

Variables	OS (n = 411)		RFS (n = 411)	
	HR (95% CI)	*P*_trend_	HR (95% CI)	*P*_trend_
Age (48 vs. <48 yrs)	0.85 (0.66–1.09)	0.19	0.80 (0.62–1.03)	0.08
Gender (Female vs. Male)	0.86 (0.66–1.13)	0.28	0.88 (0.67–1.15)	0.35
Ethnicity (Minority vs. Han)	0.89 (0.70–1.14)	0.36	0.92 (0.72–1.18)	0.52
Smoking (Yes vs. No)	1.10 (0.84–1.44)	0.50	1.10 (0.83–1.45)	0.52
Drinking (Yes vs. No)	1.20 (0.92–1.56)	0.18	1.17 (0.89–1.53)	0.26
HBsAg (Positive vs. Negative)	1.05 (0.79–1.40)	0.73	1.06 (0.79–1.42)	0.72
anti-HCV (Positive vs. Negative)	0.85 (0.57–1.27)	0.42	0.86 (0.57–1.29)	0.45
AFP (≤ 20 vs. > 20 ng/mL)	0.91 (0.71–1.18)	0.49	0.87 (0.67–1.13)	0.29
Liver cirrhosis (Yes vs. No)	1.32 (0.99–1.77)	0.06	1.36 (1.01–1.85)	0.04
tumor size (> 3 vs. ≤3 cm)	2.16 (1.67–2.78)	3.16 × 10^−9^	2.18 (1.68–2.83)	4.83 × 10^−9^
Tumor grade (High vs. Low)	1.05 (0.82–1.34)	0.71	1.06 (0.83–1.37)	0.63
BCLC stage (B-C vs. 0-A)	4.15 (3.11–5.55)	7.04 × 10^−22^	4.02 (2.98–5.43)	1.13 × 10^−19^
MVD (Positive vs. Negative)	1.96 (1.52–2.54)	2.92 × 10^−7^	1.78 (1.37–2.32)	1.60 × 10^−5^
miR-1268a expression (Low vs. High)	3.23 (2.44–4.17)	1.85 × 10^−17^	3.70 (2.78–4.76)	2.49 × 10^−19^

Abbreviations: CI, confidence interval; HR, hazards ratio, OS, overall survival; RFS, tumor reoccurrence-free survival; MVD, microvessel density.

**Table 3:** Independent prognostic factors of OS and RFS for patients with HCC by multivariate analyses.

Variables	OS (n = 411)		RFS (n = 411)	
	HR (95% CI)	*P*_trend_	HR (95% CI)	*P*_trend_
Age (48 vs. <48 yrs)	0.80 (0.61–1.03)	0.08	0.76 (0.58–0.99)	0.04
Gender (Female vs. Male)	0.87 (0.66–1.15)	0.33	0.90 (0.68–1.19)	0.46
Ethnicity (Minority vs. Han)	0.94 (0.73–1.21)	0.64	0.91 (0.70–1.18)	0.49
Smoking (Yes vs. No)	0.71 (0.39–1.32)	0.28	0.86 (0.45–1.66)	0.66
Drinking (Yes vs. No)	1.77 (0.98–3.20)	0.06	1.31 (0.70–2.46)	0.40
HBsAg (Positive vs. Negative)	1.03 (0.77–1.39)	0.83	1.00 (0.73–1.36)	0.99
anti-HCV (Positive vs. Negative)	0.86 (0.57–1.31)	0.49	0.91 (0.59–1.38)	0.65
AFP (≤20 vs. >20 ng/mL)	1.08 (0.83–1.40)	0.58	1.02 (0.78–1.34)	0.86
Liver cirrhosis (Yes vs. No)	1.19 (0.87–1.61)	0.28	1.23 (0.90–1.69)	0.19
tumor size (>3 vs. ≤3 cm)	1.90 (1.44–2.49)	4.45 × 10^−6^	1.76 (1.33–2.32)	7.27 × 10^−5^
Tumor grade (High vs. Low)	0.89 (0.68–1.15)	0.36	0.95 (0.73–1.24)	0.71
BCLC stage (B-C vs. 0-A)	3.70 (2.70–5.06)	3.71 × 10^−16^	3.44 (2.48–4.78)	1.42 × 10^−13^
MVD (Positive vs. Negative)	1.80 (1.37–2.38)	3.14 × 10^−5^	1.45 (1.09–1.93)	9.80 × 10^−3^
miR-1268a expression (Low vs. High)	2.44 (1.82–3.23)	2.37 × 10^−9^	2.86 (2.08–3.85)	4.30 × 10^−11^

Abbreviations: CI, confidence interval; HR, hazards ratio, OS, overall survival; RFS, tumor reoccurrence-free survival; MVD, microvessel density.

**Table 4:** The clinic-pathological features of HCC cases with or without TACE treatment.

Variables	Cases, n (%)	TACE treatment, n (%)		χ^2^	*P*
		No	Yes		
Total	247 (100.0)	122 (100.0)	125 (100.0)		
Age (yrs)				0.18	0.67
≤48	135 (54.7)	65 (53.3)	70 (56.0)		
>48	112 (45.3)	57 (46.7)	55 (44.0)		
Sex				21.8	0.14
Man	163 (66.0)	86 (70.5)	77 (61.6)		
Female	84 (34.0)	36 (29.5)	48 (38.4)		
Ethnicity				1.23	0.27
Han	137 (55.5)	72 (59.0)	65 (52.0)		
Zhuang	110 (44.5)	50 (41.0)	60 (48.0)		
HBsAg				0.16	0.67
Negative	62 (25.1)	32 (26.2)	30 (24.0)		
Positive	185 (74.9)	90 (73.8)	95 (76.0)		
anti-HCV				1.07	0.30
Negative	224 (90.7)	113 (92.6)	111 (88.8)		
Positive	23 (9.3)	9 (7.4)	14 (11.2)		
Smoking status				<0.001	0.99
No	176 (71.3)	87 (71.3)	89 (71.2)		
Yes	71 (28.7)	35 (28.7)	36 (28.8)		
Drinking status				0.02	0.90
No	167 (67.6)	82 (67.2)	85 (68.0)		
Yes	80 (32.4)	40 (32.8)	40 (32.0)		
AFP (ng/mL)				0.00	0.98
≤20	95 (38.5)	47 (38.5)	48 (38.4)		
>20	152 (61.5)	75 (61.5)	77 (61.6)		
Liver cirrhosis				0.47	0.49
No	51 (20.6)	23 (18.9)	28 (22.4)		
Yes	196 (79.4)	99 (81.1)	97 (77.6)		
MVD				0.06	0.81
Negative	93 (37.7)	45 (36.9)	48 (38.4)		
Positive	154 (62.3)	77 (63.1)	77 (61.6)		
Tumor grade				0.11	0.74
Low	135 (54.7)	68 (55.7)	67 (53.6)		
High	112 (45.3)	54 (44.3)	58 (46.4)		
BCLC stage				0.35	0.56
B	114 (46.2)	54 (44.3)	60 (48.0)		
C	133 (53.8)	68 (55.7)	65 (52.0)		
MiR-1268a expression				0.18	0.67
Low	171 (69.2)	86 (70.5)	85 (68.0)		
High	76 (30.8)	36 (29.5)	40 (32.0)		
